# Perception of Threat and Psychological Impact of COVID-19 among Expatriates in Makkah Region, Saudi Arabia

**DOI:** 10.3390/ijerph18126650

**Published:** 2021-06-21

**Authors:** Majed A. Algarni, Mohammad S. Alzahrani, Yasser Alatawi, Raghad A. Alasmari, Hashem O. Alsaab, Atiah H. Almalki, Abdullah A. Alhifany, Yusuf S. Althobaiti

**Affiliations:** 1 Department of Clinical Pharmacy, College of Pharmacy, Taif University, P.O. Box 11099, Taif 21944, Saudi Arabia; M.s.alzahrani@tu.edu.sa; 2 Department of Pharmacy Practice, Faculty of Pharmacy, University of Tabuk, P.O. Box 741, Tabuk 71491, Saudi Arabia; Yasser@ut.edu.sa; 3 Department of Pharmacology and Toxicology, College of Pharmacy, Taif University, P.O. Box 11099, Taif 21944, Saudi Arabia; s43602378@students.tu.edu.sa (R.A.A.); ys.althobaiti@tu.edu.sa (Y.S.A.); 4 Department of Pharmaceutics and Pharmaceutical Technology, Taif University, P.O. Box 11099, Taif 21944, Saudi Arabia; H.alsaab@tu.edu.sa; 5 Addiction and Neuroscience Research Unit, Health Science Campus, Taif University, P.O. Box 11099, Taif 21944, Saudi Arabia; ahalmalki@tu.edu.sa; 6 Department of Pharmaceutical Chemistry, College of Pharmacy, Taif University, P.O. Box 11099, Taif 21944, Saudi Arabia; 7 Department of Clinical Pharmacy, College of Pharmacy, Umm Al-Qura University, Makkah 21955, Saudi Arabia; aahifany@uqu.edu.sa

**Keywords:** COVID-19, expatriates, psychological disorders, Saudi Arabia, DASS-21, BIP-Q5

## Abstract

In the first few months of the pandemic, Makkah region reported the highest number of COVID-19 cases among all regions in Saudi Arabia. More than 80% of these reported cases were non-Saudi residents. In this study, we evaluated the perceived threat from and psychological impact of COVID-19 among non-Saudi residents of Makkah region. This was a cross-sectional analysis of data collected using a standardized self-report questionnaire. A total of 292 expatriates were included in the study, the majority of whom were non-Arabic speakers. The prevalence of self-reported depression was nearly 40%, anxiety was 32%, and stress was 43%. The findings indicated variability in the prevalence of psychological symptoms among expatriates from different ethnic backgrounds. Additionally, work environment and perceived threat were strong predictors of psychological disorders. This suggested that the perceived threat from and psychological burden of COVID-19 among non-Saudis in Makkah region is substantial. Future research should investigate the reasons behind these variations in the psychological impact of the pandemic among different ethnic groups.

## 1. Introduction

Coronavirus disease 2019 (COVID-19) is a severe respiratory disease that can lead to serious complications and death. The first case was reported in December 2019 as pneumonia of unknown origin in China [1]. In late January 2020, COVID-19 was declared a Public Health Emergency of International Concern by the World Health Organization [2]. A pandemic can affect communities and disrupt the norm, causing economic losses and depleting essential resources. There can also be an impact on individual health and well-being, leading to various emotional and psychological disorders. Psychological reactions during a pandemic play a significant role in creating outbreaks of both the disease and psychological disorders [3]. However, resources are usually not allocated to decrease the effects of a pandemic on individual mental health. Instead, mental health within communities is generally overlooked during the early stages of a pandemic, when managing the spread of infection is prioritized [3].

Over the past year, many studies have shown that the COVID-19 pandemic has negatively impacted people’s mental health. High rates of psychological symptoms have been reported in China [4], India [5], Pakistan [6], Saudi Arabia [7], Egypt [8], the Philippines [9], and Bangladesh [10] for mental health disorders such as insomnia, depression, anxiety, and stress. For instance, a nationwide Chinese study of 52,730 participants reported that 35% experienced psychological distress following the pandemic [4], while a study in Saudi Arabia found that 23.6% of participants experienced moderate-to-severe psychological symptoms [7]. Moreover, the prevalence of psychological symptoms is expected to rise, owing to many variables [11]. It is plausible that these variables could affect people’s mental health based on their sociodemographic status [12,13,14].

Evidence from previous public health emergencies indicates that socially disadvantaged groups (e.g., low-income groups, ethnic minorities) experience more health problems than socially advantaged groups [15]. Socially advantaged groups generally have access to more resources to cope with stressors caused by events such as pandemics and disasters. Thus, the disruption caused by the COVID-19 pandemic could disproportionately negatively affect the mental health of ethnic minorities and low-income individuals [16,17]. Notably, in Saudi Arabia, expatriates can be considered socially disadvantaged due to their low average income and lack of knowledge necessary to access appropriate resources to cope with the pandemic. However, there is little evidence of the impact of the COVID-19 pandemic on expatriates’ mental health in Saudi Arabia. Thus, the main objective of this study is to evaluate the psychological impact and perception of threat during the pandemic among expatriates residing in Makkah region, Saudi Arabia. The other objective is to evaluate the differences in the pandemic’s psychological impact between different expatriate ethnicities living in the Makkah region.

## 2. Materials and Methods

### 2.1. Study Design and Participants

This study utilized a cross-sectional survey design to assess the psychological impact of COVID-19 on the target population. We adopted convenience sampling to recruit expatriates living in Makkah region of Saudi Arabia during the COVID-19 pandemic from November 2020 to January 2021. The eligible study population included non-Saudi citizens aged 18 years or older who resided in Makkah region and spoke one of the following languages: Arabic, Urdu, Hindi, Bengali, or Filipino. We chose these languages because most non-Saudi residents working in Makkah region spoke at least one of them. The survey was distributed electronically, as this made it easier to circulate the questionnaire among expatriates. In addition, the Saudi government has encouraged people to minimize direct socializing as much as possible, which makes interviews and face-to-face surveys difficult. All participants provided electronic informed consent. The Scientific Research Ethics Committee of Taif University approved this study (42-0056).

### 2.2. Sample Size Calculation

We used Epi info^®^ version 7 to calculate the study sample. The study sample was calculated to be 241, assuming that 19.5% of the population would be psychologically distressed by COVID-19, based on a study conducted in of Saudi Arabia [18]. The confidence level was set at 95% and margin of error at 5%.

### 2.3. Measures

The questionnaire contained 32 items: 11 demographic questions and 21 questions from the Depression, Anxiety, and Stress Scale—21 Items (DASS-21). We collected sociodemographic data including age, gender, educational level, marital status, living arrangements (living with family), whether one worked in the medical field, job satisfaction, and monthly income.

The DASS-21 was adapted to measure the psychological impact of COVID-19 [19]. This instrument is a 21-item self-report questionnaire comprising three subscales: depression, anxiety, and stress. Each subscale contains seven items intended to measure the corresponding negative psychological state. Items are scored on a scale ranging from 0 (does not apply to me at all) to 3 (applies to me most of the time). Scores for each subscale are calculated by summing the scores for the relevant items and then multiplying this value by a factor of two. Using the recommended cut-off scores [12], each subscale was categorized into normal, mild/moderate, and severe/extremely severe. Further, each subscale was recoded into “yes” (above the cut-off score) or “no” (below the cut-off score) in relation to whether symptoms of the disorder were present in the participants.

The Brief Illness Perception Questionnaire (BIP-Q5) was also used to measure the threat participants perceived from the COVID-19 pandemic [20]. The BIP-Q5 comprises five items rated on a Likert-type scale ranging from 0 to 10. Responses were calculated to provide a summary score for the threat perceived from the pandemic. The higher the summary score, the greater the perceived threat.

### 2.4. Statistical Analysis

Descriptive analysis of sociodemographic characteristics was conducted. Mean scores were calculated for the total DASS-21 and all three subscales. Frequencies and percentages were calculated for categorical variables. Three multiple logistic regression models were built to identify factors associated with each DASS-21 subscale (i.e., depression, anxiety, and stress). Two steps were performed to build each adjusted model. First, bivariate analysis was conducted using Pearson’s chi-square test to evaluate the association between sociodemographic factors and each outcome. Second, factors that were significantly associated with the outcome (*p* < 0.05) were further analyzed and included in the adjusted model. Age and gender were included in the adjusted models, regardless of their bivariate association with the outcome. Odds ratios (ORs) and 95% confidence intervals (CIs) were calculated. All statistical analyses were conducted using SAS version 9.4.

## 3. Results

In total, 292 participants completed the survey. Table 1 shows respondents’ sociodemographic characteristics. Of the 292 respondents, 112 were non-Saudi Arabic language speakers (38%), 55 were Bengali language speakers (19%), 45 were Urdu language speakers (15%), 43 were Hindi language speakers (15%), and 37 were Filipino language speakers (13%). Additionally, 72% and 51% of the participants were men and between the ages of 18−34 years, respectively. Approximately 47% of the participants had a monthly income between 266−1600 USD.

**Figure 1:**
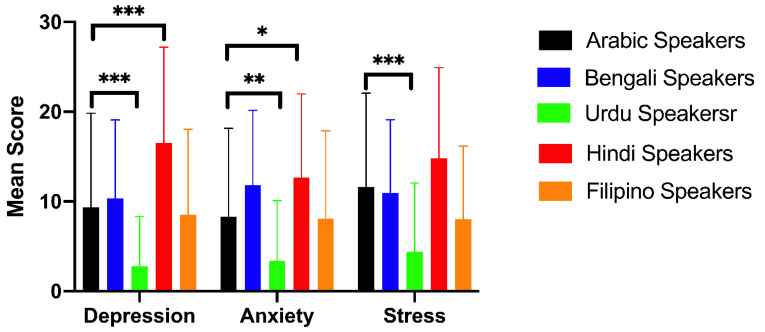
Average DASS-21 subscale scores by language speakers. * *p* < 0.05, ** *p* < 0.01, *** *p* < 0.001.

**Figure 2:**
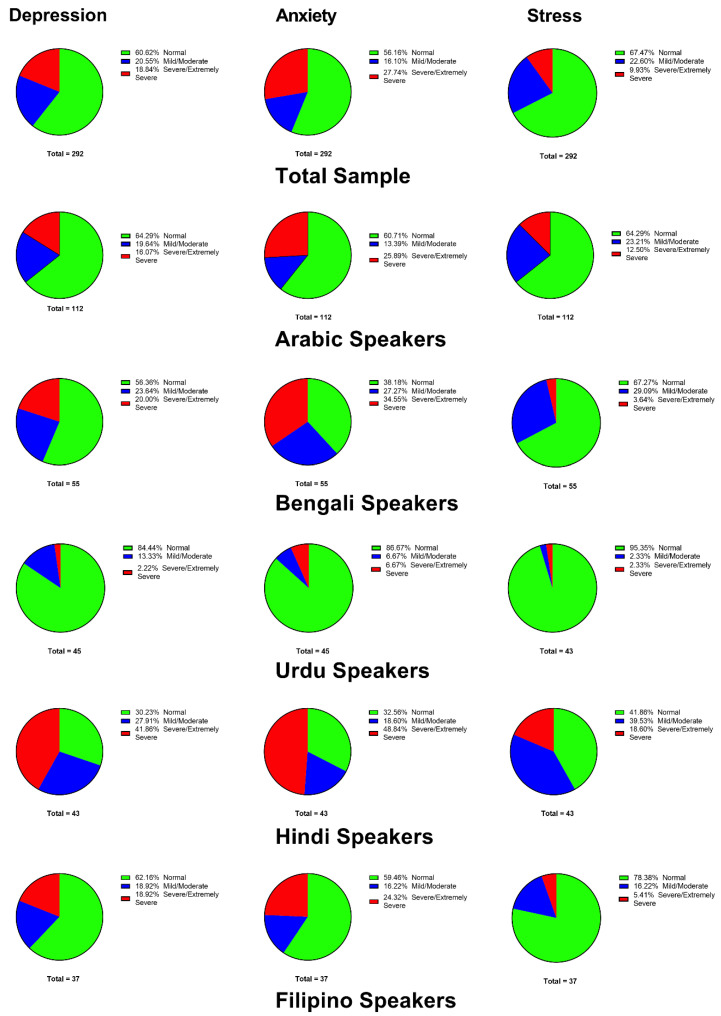
Prevalence and severity of psychological disorders by language spoken by participants.

The mean scores for the DASS-21 subscales in each group are shown in Figure 1. The results indicated that there were significant differences in mean scores for depression (F(4, 287) = 11.76, *p* < 0.0001), anxiety (F(4, 287) = 7.616, *p* < 0.0001), and stress (F(4, 287) = 8.214, *p* < 0.0001) among speakers of different languages. The post-hoc analysis found that Urdu speakers had significantly lower mean scores compared to Arabic speakers for all subscales (*p* < 0.05), while Hindi speakers had significantly higher mean scores compared to Arabic speakers for the depression and anxiety subscales (*p* < 0.0001, *p* = 0.0280). Figure 2 shows the prevalence and severity of each psychological disorder among the participants. The results showed that 39.39%, 43.84%, and 32.53% of the participants reported symptoms of depression, anxiety, and stress, respectively. Notably, Hindi speakers were more likely to report severe symptoms of depression, anxiety, and stress compared to the other groups. For instance, 41.86%, 48.84%, and 18.60% of Hindi speakers reported severe symptoms of depression, anxiety, and stress, respectively, while less than 7% of Urdu speakers reported severe symptoms for any of the DASS-21 subscales.

In comparison to male respondents, female respondents had a higher prevalence of anxiety (54.32% vs. 39.81%, *p* = 0.025) and depression (49.3% vs. 35.5%, *p* = 0.03; Table 2). Participants who lived with their families had a higher prevalence of stress than those who did not (39.82% vs. 27.68%, *p* = 0.03). Respondents who worked in the medical field had a significantly higher prevalence of anxiety (58.95% vs. 37.31%, *p* = 0.0005) and a higher prevalence of both depression (53.68% vs. 32.12%, *p* = 0.0004) and stress (43.16% vs. 27.98%, *p* = 0.1). Respondents who said that they were satisfied with their jobs during the pandemic had a significantly lower prevalence of anxiety compared to those who were not satisfied with their jobs (35.71% vs. 55.74%, *p* = 0.0007), lower prevalence of depression (29.76% vs. 53.28%, *p* ≤ 0.0001), and lower prevalence of stress (26.2% vs. 41.8%, *p* = 0.005). People in the lowest income category had a significantly higher prevalence of anxiety than those in the middle- and higher-income groups (54.79% vs. 35% and 50%, *p* = 0.01).

The results showed that the perception of threat among participants on most of the BIP-Q5 items was significantly correlated with the DASS-21 subscales (Table 3). Specifically, a higher perception of threat regarding the consequences of the COVID-19 pandemic in one’s life was positively correlated with higher scores for depression, anxiety, and stress (*r* = 0.29, *p* < 0.0001; *r* = 0.32, *p* < 0.0001; *r* = 0.31, *p* < 0.0001, respectively). As shown in Table 4, the multivariate analysis indicated that Hindi speakers were four to six times more likely to report symptoms of depression, anxiety, and stress (OR = 5.99, 95% CI: 2.53–14.17; OR = 5.23, 95% CI: 2.09–13.08; OR = 4.37, 95% CI: 1.80–10.70, respectively), while Filipino and Urdu speakers were less likely to report symptoms of stress (OR = 0.19, 95% CI: 0.05–0.86; OR = 0.22, 95% CI: 0.06–0.85, respectively). Furthermore, perceived threat and job satisfaction were also significantly associated with all DASS-21 subscales.

## 4. Discussion

This study found a high prevalence of psychological symptoms among expatriates in Saudi Arabia during the COVID-19 pandemic. This highlights the significant impact of the pandemic on people’s mental health, especially among expatriates. Over a third of the participants reported symptoms of depression, anxiety, and stress. This was in line with some previous research, such as one study that examined the prevalence of stress among expatriates in the Al-Ahsa region and found that 31% reported severe symptoms of stress during the travel ban [21]. Another recent study found that 19.7% of expatriates reported symptoms of depression [22].

Additionally, the present study found that there are variations in the prevalence of psychological symptoms among speakers of different languages. For instance, Indian expatriates showed a higher chance of experiencing anxiety, depression, and stress than non-Saudi Arabic speakers. Although it is not clear why Indians have a higher chance of becoming anxious and depressed, one explanation could be the language barrier. However, speakers of other languages showed lower odds compared to non-Saudi Arabic speakers. A study conducted in Bahrain, a GCC country, found that Pakistanis had higher rates of stress, anxiety, and depression than Indians; however, Indians were overrepresented in the sample [23]. Another study conducted on Indian expatriates in the Middle East found that psychological stress in this population was significantly higher compared to the pre-COVID-19 period [24]. A recent study conducted in Saudi Arabia that examined stress due to travel bans among expatriates found that those from India experienced the highest levels of stress [21]. A study conducted with male expatriates in Saudi Arabia found that Indian people experienced more depressive symptoms than Pakistanis and Egyptians [22]. The differences among ethnic groups found in the present study may be due to different sociocultural factors or actual differences in the psychological burden on different ethnic groups residing in Saudi Arabia. This highlights two important points. First, it is necessary to give further attention to the mental health of expatriates, even after the COVID-19 pandemic, to understand the root of this issue. Second, stronger policy decisions are needed that aim to provide expatriates with access to appropriate mental health care.

In a study that was published last year and was conducted on the general Saudi population, [7] the authors found that 70% of the sample was in the normal range of the stress and anxiety subscales, and 60% was in the normal range of the depression subscale. We can see that there is a clear difference between these findings and the findings of this study, which may imply that expatriates in Saudi Arabia have suffered psychologically more than Saudi citizens.

This study also found that working in the medical field, which included approximately one-third of the 292 respondents (33%), was associated with depression and stress. In line with this finding, a cross-sectional study of healthcare workers in Saudi Arabia and Egypt reported high prevalence rates for depression (69%), anxiety (59%), and stress (56%) during the COVID-19 pandemic [25]. In contrast, the prevalence rates for depression, anxiety, and stress were shown to be very low among healthcare workers in Singapore (8%, 11%, and 6%, respectively) [26]. These relatively low rates of psychological symptoms compared to many countries in the Middle East could be attributed to the high preparedness and rigorous infection control measures in Singapore that were enacted following the SARS epidemic. Furthermore, the results also suggested that work satisfaction is associated with lower levels of psychological symptoms. Similarly, one study in Saudi Arabia found that job dissatisfaction among nurses was associated with mild-to-moderate depression [27].

This study found that perceived threat from the COVID-19 pandemic was associated with an increased risk for depression, anxiety, and stress. These findings are consistent with previous evidence which indicated that a perceived threat increases the risk of stress and may subsequently lead to the development of psychological disorders [28,29]. For instance, a study in Spain found that the perception of threat was associated with anxiety and depression [30].

This study has some limitations. First, the use of convenience sampling and the small sample size might limit the generalizability of the results. Further investigation is required to include a more diverse sample that represents the expatriate population in Saudi Arabia. Second, we did not have sufficient resources to validate these instruments in different languages. However, these instruments have been validated and translated into many languages, including those used in this study [31].

## 5. Conclusions

Almost half the population of Makkah region is composed of non-Saudi residents. In this study, we explored perceived threat from and the psychological impact of the COVID-19 pandemic among expatriates residing in Saudi Arabia. The findings indicated a high prevalence of depression, anxiety, and stress among expatriates. Moreover, it was found that there is variability in the prevalence of psychological symptoms among expatriates from different ethnic backgrounds. Additionally, work environment and perceived threat were strong predictors of psychological symptoms. Further research is needed to better understand differences in psychological symptoms among expatriates working in Saudi Arabia.

## Figures and Tables

**Table 1:** Sociodemographic characteristics of the respondents (N = 292).

Characteristic	N (%)
Male	211 (72%)
Married	161 (56%)
Age	
18−34 years	149 (51%)
35−44 years	83 (28%)
≥45 years	60 (21%)
Education level	
Did not finish high school	129 (45%)
High school or higher	158 (55%)
Monthly income	
<1000 SR ≈ <266 USD	73 (25%)
1000 SR–6000 SR ≈ 266–1600 USD	137 (47%)
>6000 SR ≈ >1600 USD	80 (28%)
Living with family	113 (39%)
Working in the medical field	95 (33%)
Language	
Non-Saudi Arabic speakers	112 (38%)
Bengali speakers	55 (19%)
Urdu speakers	45 (15%)
Hindi speakers	43 (15%)
Filipino speakers	37 (13%)
Satisfied with work	
Yes	168 (58%)
No	124 (42%)

**Table 2:** Bivariate analysis of sociodemographic factors and psychological disorders.

Characteristic	Anxiety %	p *	Depression %	p *	Stress %	p *
Gender						
Male	39.81	0.025	35.5	0.03	30	0.2
Female	54.32		49.3		38	
Age						
18–34	42.28	0.006	38.9	0.2	32.89	0.03
35–44	56.63		45.8		40.96	
≥45	30		31.7		20	
Living with family						
Yes	47.79	0.27	44.25	0.17	39.82	0.03
No	41.24		36.16		27.68	
Working in the medical field						
Yes	58.95	0.001	53.68	<0.001	43.16	0.01
No	37.31		32.12		27.98	
Monthly income						
<1000 SR	54.79	0.01	43.8	0.55	39.73	0.13
1000–6000 SR	35		36.5		27	
>6000 SR	50		41.25		36.25	
Satisfied with job						
Yes	35.71	0.001	29.76	<0.001	26.2	0.005
No	55.74		53.28		41.8	
Language						
Arabic	39.29	<0.001	35.71	<0.001	35.7	<0.001
Bengali	61.82		43.64		32.7	
Urdu	13.33		15.56		8.9	
Hindi	67.44		69.77		58	
Filipino	40.54		37.84		21.6	

* *p*-values produced by Pearson’s chi-square test.

**Table 3:** Correlations between BIPQ-5 items and DASS-21 items.

BIPQ-5	DASS-21					
	Depression		Anxiety		Stress	
	*r*	*p*	*r*	*p*	*r*	*p*
Consequences	0.29	<0.001	0.32	<0.001	0.31	<0.001
Timeline	0.27	<0.001	0.35	<0.001	0.31	<0.001
Identity	0.12	0.034	0.19	0.001	0.09	0.090
Concern	0.09	0.104	0.18	0.003	0.10	0.077
Emotional response	0.24	<0.001	0.33	<0.001	0.19	0.001
Summary score	0.27	<0.001	0.36	<0.001	0.27	<0.001

Abbreviations: BIPQ-5: 5-item brief illness perception questionnaire; DASS-21: Depression Anxiety Stress Scale; *r*: Pearson correlation coefficient.

**Table 4:** Multivariate logistic analysis results for depression, anxiety, and stress.

Predictors	Adjusted OR	95% CI	*p*
Factors associated with anxiety			
Filipino speakers (vs. Arabic speakers)	0.28	0.08–0.88	0.006
Hindi speakers (vs. Arabic speakers)	5.23	2.09–13.08	<0.001
35−44 years old (vs. >45 years old)	2.92	1.23–6.89	0.018
Income 1000−6000 SR (vs. <1000 SR)	0.43	0.21–0.89	0.004
Satisfied with job (yes vs. no)	0.39	0.21–0.72	0.003
Perceived threat from COVID-19	1.07	1.04–1.10	<0.001
Factors associated with depression			
Hindi speakers (vs. Arabic speakers	5.99	2.53–14.17	<0.001
Satisfied with job (yes vs. no)	0.37	0.21–0.66	0.001
Perceived threat from COVID-19	1.05	1.03–1.08	<0.001
Factors associated with stress			
Living with family (yes vs. no)	2.53	1.23–5.27	0.012
Hindi speakers (vs. Arabic speakers	4.37	1.8–10.7	<0.001
Filipino speakers (vs. Arabic speakers)	0.19	0.05–0.68	0.005
Urdu speakers (vs. Arabic speakers)	0.22	0.06–0.85	0.021
Working in medical field (yes vs. no)	2.02	1.03–3.98	0.041
Perceived threat from COVID-19	1.04	1.02–1.07	0.001

## Data Availability

Data are available upon reasonable request to the corresponding author.
